# Exploring the Interrelationships between Public Health, Fiscal Decentralization, and Local Government Debt in China

**DOI:** 10.3390/healthcare11142103

**Published:** 2023-07-24

**Authors:** Mingyao Cao, Keyi Duan, Mingyu Cao, Haslindar Ibrahim

**Affiliations:** 1 School of Management, Universiti Sains Malaysia, Gelugor 11800, Penang, Malaysia; 2 Lee Kuan Yew School of Public Policy, National University of Singapore, Singapore 259772, Singapore; 3 Department of City and Regional Planning, University of North Carolina at Chapel Hill, Chapel Hill, NC 27514, USA; 4 UCD College of Business, University College Dublin, Belfield, D04 V1W8 Dublin, Ireland

**Keywords:** public health, fiscal decentralization, local government debt, healthcare policy, fiscal policy

## Abstract

This paper investigates the interrelationships among local government debt, fiscal decentralization, and public health. The investigation begins by constructing a theoretical model to analyze the inherent connections between these variables. Subsequently, an empirical analysis is conducted using data from China between 2015 and 2021. The findings demonstrate a bidirectional relationship between fiscal decentralization, local government debt, and public health. Specifically, it is observed that an increase in local government debt has adverse effects on both fiscal decentralization and public health, while fiscal decentralization has a positive impact on public health. These insights are consistently validated through rigorous regression methodologies, affirming the robustness and significance of these relationships.

## 1. Introduction

Health, as a fundamental social and economic right, represents a primary responsibility for governments worldwide [1]. The post-pandemic era has brought to the forefront the crucial equilibrium between economic development and public health, especially in light of the vulnerabilities exposed by the COVID-19 pandemic within global healthcare systems. China serves as a noteworthy illustration, wherein less developed regions face the difficulties associated with uneven economic development, leading to underfunded healthcare infrastructures [2]. This imbalance significantly burdens public health systems at various administrative levels.

In response, China’s central government has taken steps to bolster public health development through the augmentation of fiscal budgets and healthcare system reforms [3]. Nevertheless, local governments, often lacking in the necessary financial and human resources, struggle to support public health initiatives efficiently. Evidence from nations adopting decentralized health systems reveals discord in the implementation of healthcare policies between central and local governments [4,5,6]. Ascertaining the unique challenges in China’s public health sector, research has highlighted that discrepancies in local economic development give rise to inequalities in public health progression. Economically disadvantaged regions frequently confront underinvestment in their environment and public health sectors [7,8]. On the other hand, regions demonstrating a higher degree of fiscal decentralization, with autonomy in fund allocation, can dedicate more resources to healthcare services [9]. This scenario often culminates in better-funded, resilient healthcare systems. However, regions burdened by excessive local government debt grapple with an impetus to prioritize debt management [10], often leading to a reduction in funding for healthcare infrastructure and services, thereby compromising the quality and accessibility of public health services.

Examining the finance mechanisms, local governments in China play a pivotal role in financing public health infrastructure [11]. For the central government, entrusted with financial resource allocation, it is imperative to distribute funds judiciously to minimize regional disparities in economics and public health, and regulate government debt. While the central government zeroes in on macro healthcare and economic development [12], local governments, bound by a performance evaluation framework predominantly oriented towards economic growth, prioritize short-term regional economic expansion [13]. Building a robust healthcare system, a long-term endeavor, often takes a backseat, leading to constraints in fiscal resource allocation for economic growth.

In the lens of fiscal decentralization, China is recognized as one of the most fiscally decentralized nations worldwide [14]. Fiscal decentralization boosts central fiscal income, empowering the central government to orchestrate disparate regional economic developments [15]. Concurrently, local governments bear the responsibility for funding infrastructure, healthcare, education, and public service development. This responsibility often creates a divergence between fiscal power and accountability, compelling local governments to incur substantial debts to cover funding deficits. Existing literature suggests that fiscal decentralization may heighten the risk of government debt default and excessive transfers from the central government can also inflate local government debt [16]. However, the discourse on the interconnectedness between fiscal decentralization, government debt, and public health remains scant, with the operative mechanism of how fiscal decentralization and government debt impact public health largely unexplored.

To contribute to this body of knowledge, we conducted empirical and model analyses using data from 31 Chinese provinces spanning the period from 2015 to 2021. Our findings indicate that a rise in local government debt leads to a reduction in fiscal decentralization, attributable to resource reallocation for debt service. Conversely, fiscal decentralization induces an increase in local debt when expenditures exceed revenues. An enhancement in public health corresponds with increased fiscal decentralization expenditure, denoting a more substantial resource allocation towards health. Simultaneously, as public health conditions improve, there is a recorded decline in fiscal decentralization income, indicative of reduced reliance on fiscal decentralization for income generation. Moreover, the results unveil that an upswing in government debt results in curtailed investments in public health, potentially exacerbating health conditions. In contrast, public health improvements trigger an increase in government debt, which is likely a consequence of extensive investments in health infrastructure and services.

## 2. Theoretical Analysis and Literature Review

### 2.1. Theoretical Analysis

In this study, the model proposed by Holmstrom and Milgrom [17] is refined. In our framework, the central government is considered the principal, while the local governments act as agents. The primary responsibilities of local governments include managing local government debt and improving public health infrastructure. Two effort allocations, $x_{D}$ and $x_{H}$, are defined, where $x_{D}$ signifies the effort allocated to local government debt control and $x_{H}$ represents the effort allocated to the development of public health infrastructure. The total output of the local government, denoted as Z, is assumed to follow a Cobb–Douglas function, which is a function of the efforts expended in managing local government debt $\left(x_{D}\right)$ and enhancing public health infrastructure ($x_{H}$), along with error terms $\varepsilon_{D}\;\mathrm{and}\;\varepsilon_{H}$. This relationship is represented by Equation (1) as follows:

(1) $$Z=A\times \left(x_{D}^{\alpha }\right)\times \left(x_{H}^{\beta }\right)$$

An effort function, denoted as $E\left(x_{D},x_{H}\right)$, is introduced to quantify the cost associated with these efforts for the regional government. It is anticipated that this cost will be positive and increase with the level of effort. Assuming a constant risk aversion bias (*ρ*) among regional governments, the risk cost is calculated as $0.5\rho \left(\sigma_{D}^{2}x_{D}^{2}+\sigma_{H}^{2}x_{H}^{2}\right)$, where $\sigma_{D\;}^{2}\mathrm{and}\;\sigma_{H}^{2}$ represent the variances of the efforts.

The model further incorporates conditions for Participation (CR) and Incentives (IN), which are formulated as Equations (2) and (3), respectively, as follows:

(2) $$\left(CR\right)\alpha +\beta^{T}Z-0.5\rho \left(\sigma_{D}^{2}x_{D}^{2}+\sigma_{H}^{2}x_{H}^{2}\right)-E\left(x_{D},x_{H}\right)>P$$

(3) $$\left(IN\right)\left(x_{D},x_{H}\right)\in arg\;maxP\left(Z\right)-0.5\rho \left(\sigma_{D}^{2}x_{D}^{2}+\sigma_{H}^{2}x_{H}^{2}\right)-E\left(x_{D},x_{H}\right)$$

Upon differentiation of the aforementioned equations, the Certainty Equivalent (CE) of the national government is derived and found to be zero. Equations (4) and (5) can be expressed as follows:

(4) $$\frac{\partial CE}{\partial Z_{D}}=\alpha \times A\times {\left(x_{D}+\varepsilon_{D}\right)}^{\alpha -1}\times {\left(x_{H}+\varepsilon_{H}\right)}^{\beta }-\frac{\partial E}{\partial Z_{D}}=0$$

(5) $$\frac{\partial CE}{\partial Z_{H}}=\beta \times A\times {\left(x_{D}+\varepsilon_{D}\right)}^{\alpha }\times {\left(x_{H}+\varepsilon_{H}\right)}^{\beta -1}-\frac{\partial E}{\partial Z_{H}}=0$$

By solving these equations, the optimal values of α and β are obtained, as shown in Equations (6) and (7):

(6) $$\alpha \times A\times {\left(x_{D}+\varepsilon_{D}\right)}^{\alpha -1}\times {\left(x_{H}+\varepsilon_{H}\right)}^{\beta }=\frac{\partial E}{\partial Z_{D}}$$

(7) $$\beta \times A\times {\left(x_{D}+\varepsilon_{D}\right)}^{\alpha }\times {\left(x_{H}+\varepsilon_{H}\right)}^{\beta -1}=\frac{\partial E}{\partial Z_{H}}$$

The maximum value is determined by converting these constraints into IR and CR constraints. Equation (8) can be expressed as follows:

(8) $$max\alpha +\beta^{T}Z-P-0.5\rho \left(\sigma_{D}^{2}x_{D}^{2}+\sigma_{H}^{2}x_{H}^{2}\right)-E{\left(x_{D},x_{H}\right)}^{\prime \prime }{s.t.}^{\prime \prime }\;\alpha +\beta^{T}Z-0.5\rho \left(\sigma_{D}^{2}x_{D}^{2}+\sigma_{H}^{2}x_{H}^{2}\right)-E\left(x_{D},x_{H}\right)\geq P,\;\;\alpha \times A\times {\left(x_{D}+\varepsilon_{D}\right)}^{\alpha -1}\times {\left(x_{H}+\varepsilon_{H}\right)}^{\beta }=\frac{\partial E}{\partial Z_{H}}$$

Further guidance obtained Equations (9) and (10):

(9) $$\frac{1-\alpha -\alpha \cdot A\cdot {\left(x_{D}+\varepsilon_{D}\right)}^{\alpha -1}\cdot {\left(x_{H}+\varepsilon_{H}\right)}^{\beta }\cdot \left(\frac{\partial^{2}E}{\partial Z_{D}^{2}}\right)}{-\beta \cdot A\cdot {\left(x_{D}+\varepsilon_{D}\right)}^{\alpha }\cdot {\left(x_{H}+\varepsilon_{H}\right)}^{\beta -1}\cdot \left(\frac{\partial^{2}E}{\partial Z_{D}\partial Z_{H}}\right)}=0$$

(10) $$\frac{1-\beta -\beta \cdot A\cdot {\left(x_{D}+\varepsilon_{D}\right)}^{\alpha }\cdot {\left(x_{H}+\varepsilon_{H}\right)}^{\beta -1}\cdot \left(\frac{\partial^{2}E}{\partial Z_{H}^{2}}\right)}{-\alpha \cdot A\cdot {\left(x_{D}+\varepsilon_{D}\right)}^{\alpha -1}\cdot {\left(x_{H}+\varepsilon_{H}\right)}^{\beta }\cdot \left(\frac{\partial^{2}E}{\partial Z_{D}\partial Z_{H}}\right)}=0$$

Upon solving Equations (9) and (10), the coefficients α and *β* are obtained, which represent the weight assigned by the central government to debt control (α) and public health infrastructure development (β) within their incentive scheme.

Equations (9) and (10) are solved, yielding the following results:

(11) $$\alpha =\frac{1-\beta \cdot A\cdot {\left(x_{D}+\epsilon_{D}\right)}^{\alpha -1}\cdot {\left(x_{H}+\epsilon_{H}\right)}^{\left(\beta -1\right)}\cdot \left(\frac{\partial^{2}E}{\partial Z_{D}\partial Z_{H}}\right)}{1+A\cdot {\left(x_{D}+\epsilon_{D}\right)}^{\alpha -1}\cdot {\left(x_{H}+\epsilon_{H}\right)}^{\beta }\cdot \left(\frac{\partial^{2}E}{\partial Z_{D}^{2}}\right)}$$

(12) $$\beta =\frac{1-\alpha \cdot A\cdot {\left(x_{D}+\epsilon_{D}\right)}^{\alpha -1}\cdot {\left(x_{H}+\epsilon_{H}\right)}^{\beta }\cdot \left(\frac{\partial^{2}E}{\partial Z_{D}\partial Z_{H}}\right)}{1+A\cdot {\left(x_{D}+\epsilon_{D}\right)}^{\alpha }\cdot {\left(x_{H}+\epsilon_{H}\right)}^{\left(\beta -1\right)}\cdot \left(\frac{\partial^{2}E}{\partial Z_{H}^{2}}\right)}$$

where:

$$E_{DD}=\frac{\partial^{2}E}{\partial Z_{D}^{2}},\;\;E_{DH}=\frac{\partial^{2}E}{\partial Z_{D}\partial Z_{H}},\;\;E_{HH}=\frac{\partial^{2}E}{\partial Z_{H}^{2}}$$

The variable $E_{DH}$ captures the interaction between debt management and the development of public health infrastructure. A positive value of $\;E_{DH}$ indicates a discrepancy between the actions of the local administration and the responsibilities delegated by the federal government in terms of debt management and public health infrastructure development. A value of zero for $\;E_{DH}$ suggests that these two aspects operate independently. Conversely, a negative value of $E_{DH}$ indicates that the activities of the local government related to debt control and public health infrastructure are aligned with the directives issued by the central authority. Equation (13) provides the derived result for $E_{DH}$:

(13) $$\alpha =\frac{1-\frac{E_{DD}}{E_{DH}}+\frac{1}{A{\left(x_{D}+\varepsilon_{D}\right)}^{\alpha -1}{\left(x_{H}+\varepsilon_{H}\right)}^{\beta }E_{HH}}}{\frac{1}{A{\left(x_{D}+\varepsilon_{D}\right)}^{\alpha -1}{\left(x_{H}+\varepsilon_{H}\right)}^{\beta }E_{HH}}+\frac{{\left(x_{D}+\varepsilon_{D}\right)}^{\alpha }E_{DD}}{{\left(x_{H}+\varepsilon_{H}\right)}^{\beta }E_{HH}}+1+{\left(x_{D}+\varepsilon_{D}\right)}^{\alpha }\left(E_{DD}-\frac{E_{DH}^{2}}{E_{HH}}\right)}$$

Simplifying further, as show in Equation (14):

(14) $$\alpha =\frac{1-\frac{E_{DD}}{E_{DH}}}{1+{\left(x_{D}+\varepsilon_{D}\right)}^{\alpha }\times \left(E_{DD}-\frac{E_{DH}{}^{2}}{E_{HH}}\right)}$$

The model accentuates the endeavors undertaken by local governments to proficiently manage government debt and boost public health infrastructure. The overall output (Z) manifests from the interaction of these dual efforts. A shift towards more rigorous debt control by local governments can lead to a contraction in resources available for public health infrastructure enhancement. This situation reveals a potential compromise between these two critical priorities, compelling the central government to negotiate a balanced approach, ensuring proficient management of both areas. The concept of fiscal decentralization refers to the degree of fiscal authority transitioned from the central government to local entities. In this model, the role of managing government debt and fostering public health infrastructure development is assigned to local governments. An escalation in fiscal decentralization could grant local governments increased autonomy and resources, which could be allocated towards public health infrastructure advancement. With respect to government debt and fiscal decentralization: a surge in fiscal decentralization may confer additional authority and accountability on local governments in managing their debt. Heightened fiscal decentralization could equip local governments with the capacity to more effectively respond to local needs, potentially optimizing debt management and indirectly favoring the health sector. This interaction underlines the intricate and nuanced dynamics among fiscal decentralization, government debt, and public health, necessitating careful consideration and policy development in these interconnected domains.

Drawing from the model, the following hypotheses are proposed:

**H1:**  *Fiscal Decentralization and Local Government Debt: A rise in local government debt results in reduced fiscal decentralization due to resource diversion for debt service. On the flip side, fiscal decentralization amplifies local debt when expenditure outstrips revenue*.

**H2:**  *Public Health and Fiscal Decentralization: Improved public health corresponds with increased fiscal decentralization expenditure and potential reductions in fiscal decentralization income as health conditions ameliorate.*

**H3:**  *Government Debt and Public Health: An inverse relationship between government debt and public health is proposed, where increased debt leads to curtailed public health investments. Concurrently, improved public health is associated with escalated government debt*.

### 2.2. Literature Review

#### 2.2.1. Relationship between Fiscal Decentralization and Local Government Debt

In the literature review, a detailed exploration of the complex nexus between fiscal decentralization and local government debt is undertaken by numerous studies. Consistency with the conclusions drawn by Lam, et al. [18] and Jia, et al. [19] is observed in the findings, which suggest that escalated spending due to increased fiscal autonomy leads to a rise in local government borrowing and debt. However, the intensity of this effect is noted to be dependent on institutional factors, such as the stringency of fiscal rules and the political environment. On the other hand, efficient local government spending, potentially offsetting some debt impacts, is suggested to be fostered by fiscal decentralization, as posited by Baskaran [20].

Conversely, the assertion that fiscal decentralization can be curtailed by heightened local government debt is supported by studies carried out by Nguyen and Anwar [21], Li and Lin [22], and McCauley and Ma [23]. Their research suggests a response mechanism where central governments react to escalating local debt levels by tightening fiscal control, leading to decreased decentralization. This effect’s intensity is influenced by the scale of inter-governmental fiscal transfers and the willingness of the central government to rescue indebted local governments. The intricate interrelationship between local government debt and fiscal decentralization is highlighted in this body of literature, emphasizing the need for further research to clarify the exact dynamics at work.

The theory of fiscal federalism is relevant in this context, postulating that local governments have better positioning to understand and cater to the unique needs of their constituencies [24]. Expectations of potential bailouts from the central government, coupled with increased fiscal autonomy, tend to drive local governments to escalate spending to meet local demands, often leading to borrowing [25]. This borrowing to finance expenditures results in debt accumulation and, consequently, a build-up of local government debt. In response to rising local government debt levels that could jeopardize fiscal stability, the central governments may reassert fiscal control [26], leading to a reduction in the fiscal autonomy of local governments [27]. This suggests a cyclical theoretical process: fiscal decentralization may trigger an increase in local government debt and this debt accumulation may, in turn, provoke a decrease in fiscal decentralization. This cycle could repeat, creating a fluctuating pattern of increasing fiscal decentralization and growing debt burden.

#### 2.2.2. Fiscal Decentralization and Public Health

A significant body of research suggests a negative correlation between fiscal decentralization and public health outcomes. Rotulo, et al. [28] and Arends [29], for instance, have found that increased levels of fiscal decentralization often lead to disparities in health outcomes. This is attributed to the varying abilities of local governments to manage health resources and services effectively. They argue that, particularly in less affluent regions, local governments may lack the requisite expertise and resources to manage health services efficiently, resulting in subpar health outcomes. Similarly, studies by Jin and Sun [30] and Wang and Nayak [31] suggest that fiscal decentralization can result in inefficiencies and coordination problems among local governments, culminating in poorer health outcomes. They emphasize that the fragmentation of health services and the absence of uniform standards associated with fiscal decentralization can compromise the quality and accessibility of healthcare.

Conversely, there is also research demonstrating a positive correlation between fiscal decentralization and public health outcomes. For example, studies by Jin and Zou [32] and Asfaw, et al. [33] indicate that, when local governments have greater control over resources, they can customize health policies and interventions to meet local needs more effectively, thereby enhancing health outcomes. They argue that fiscal decentralization can lead to more localized and responsive health policies, facilitating a more efficient allocation of resources and better health outcomes. Furthermore, research by Khaleghian [34] and Wang, He, and Niu [15] shows that fiscal decentralization can stimulate increased public health investment. They posit that local governments are encouraged to allocate more resources toward health services in response to local demand. This investment can drive the development and improvement of health infrastructure and services, resulting in improved health outcomes.

These diverse findings indicate that the impact of fiscal decentralization on public health is highly context-dependent and influenced by multiple factors, including government income and expenditure. Yet, few researchers have investigated the relationship between fiscal decentralization and public health within different models of fiscal decentralization. Fiscal decentralization of income, where local governments have control over their revenue sources, can have a complex impact on public health outcomes. On the other hand, fiscal decentralization of expenditure, where local governments control how their funds are spent, has a different impact on public health outcomes. This area presents a fertile ground for future research to explore and understand these complex dynamics more fully.

#### 2.2.3. Local Government Debt and Public Health

The fiscal stress theory, a crucial concept in the field, proposes that rising government debt can cause budgetary pressures, necessitating austerity measures, potentially including cuts to public health spending [35]. This theory is supported by the works of Fanelli, et al. [36] and Stuckler, et al. [37], who argue that increased debt can limit resources for public health infrastructure and services, potentially deteriorating public health outcomes. Concurrently, public finance literature suggests that public health improvements often require substantial investments in health infrastructure, preventive measures, and treatment programs. This could lead to an increase in government debt, especially if the government is the primary provider or funder of these services, and if these expenditures are not offset by other revenue sources [38,39].

Theoretical analyses and empirical results drawn from the existing literature indicate a bidirectional relationship between government debt and public health. While increasing government debt can adversely impact public health, advancements in public health can conversely lead to a rise in government debt. Given the significant implications of both government debt and public health for societal wellbeing and economic stability, further research to unravel this complex relationship is crucial. Such an understanding will assist in the development of policy responses that can effectively manage the involved trade-offs, and strike a balance between fiscal sustainability and public health outcomes.

#### 2.2.4. Public Health Expenditure

According to the WHO’s report [40] in 2021, public health expenditure is comprised of several distinct components, including preventive care, curative care, health infrastructure, health research and development, and administrative costs. The 2021 China Health and Health Development Statistical Bulletin reveals that government and social public health expenditures accounted for 72.6% of total health expenditures, illustrating the significant role of government funding in China’s health expenditure.

Each aspect of public health is distinctly influenced by the extent and strategy of fiscal decentralization. It is plausible that fiscal decentralization might afford local governments more control over their budgets, which could lead to increased investments in priority sectors such as preventive care or health infrastructure. Nevertheless, decentralization could potentially accentuate disparities if wealthier regions possess the capability to dedicate more resources to health services compared to their less prosperous counterparts. This underlines the complex interrelation between fiscal decentralization and health expenditures [41]. Prior studies emphasize that the impact of fiscal decentralization varies across different types of health expenditures. Huang, et al. [42] documented a positive correlation between fiscal decentralization and increased investments in health infrastructure and public health programs, respectively. Conversely, Wang, et al. [43] argue that escalated fiscal decentralization magnifies regional disparities, especially in preventative health services. Sun and Andrews [9] provides a unique perspective, suggesting that fiscal decentralization improves public health efficiency and service expenditure. Although our investigation primarily concentrates on the effect of fiscal decentralization on provincial fiscal expenditure and income, the insights derived from these previous studies provide an indispensable context. Importantly, they indicate the necessity for further research into the mechanisms influencing the relationship between fiscal decentralization and varied health expenditures in China.

## 3. Research Design and Data

### 3.1. Endogenous Variables

#### 3.1.1. Fiscal Decentralization

Our research methodology builds upon the established framework for fiscal decentralization proposed by the Organization for Economic Co-operation and Development (OECD). This comprehensive approach entails broad measures of government expenditure and revenue, inclusive of total spending, revenue, and intergovernmental transfers. This methodology offers an extensive macroeconomic perspective of fiscal decentralization and has been extensively deployed in previous research, owing to its efficacy in interpreting economic discrepancies across countries.

However, the aim of our study is to delve into the intricacies of fiscal decentralization within distinct provinces, thereby requiring the incorporation of additional dimensions. To this end, we introduce two pivotal variables: fiscal decentralized expenditure (FDE) and fiscal decentralized income (FDI). Both FDE and FDI serve to provide an encompassing overview of local governmental public sector activities. Their merit as robust measures of fiscal decentralization within the context of China has been corroborated by the research conducted by Zhang, et al. [44], Zhang, Zhou, Wang, Ding, and Zhao [10], and Cheng and Zhu [45]. As has been underscored by the aforementioned scholars, the utilization of the ratio of provincial consolidated expenditure per capita to national consolidated expenditure per capita for the computation of FDE and FDI is a widely recognized research indicator.

#### 3.1.2. Government Debt

With the implementation of the revised Budget Law of the People’s Republic of China on 1 January 2015, local governments have been permitted solely to issue government bonds as a means to accrue debt. Specifically, Article 35, paragraph 3 of the Budget Law unequivocally mandates that local entities must not amass debt through any alternative mechanisms. Special bonds, which are issued by provincial governments for public welfare projects with certain income, undertake to repay the principal and interest within a stipulated timeframe using government funds or special revenues corresponding to those public welfare projects. Since 2015, these special bonds have constituted the primary form of bonds issued by local governments. Therefore, in this paper, special bonds are deployed as a representation of local government debt.

#### 3.1.3. Public Health

Perinatal mortality is an effective representation of public health levels, and it is a significant metric for assessing public health and the health status of the nation [46,47,48,49]. The World Health Organization (2006) [50] defines perinatal mortality as the “number of stillbirths and deaths in the first week of life per 1000 total births”. This metric is not only a crucial indicator of maternal care, maternal health, and nutrition, but it also reflects the efficacy of healthcare and public health measures [51]. Additionally, it mirrors socio-economic development [52]. Thus, in this paper, perinatal mortality is utilized as an indicator of public health.

### 3.2. Exogenous Variables

Fiscal transparency is incorporated as an exogenous variable; fiscal transparency can enhance economic activity by cultivating an atmosphere of trust; it can also potentially affect provincial fiscal conditions. Density of medical institutions is deemed an exogenous variable. An increased density of medical institutions may foster better healthcare access, thereby enhancing overall population health. Concurrently, it might also impact medical staff density as the number of healthcare facilities in a region can shape the distribution and availability of medical staff. Medical staff density is also selected as an exogenous variable. The accessibility and density of medical staff could directly influence the quality of healthcare services, thereby affecting the overall health of the population. Gross domestic product at the provincial level is selected as an exogenous variable as well. The level of GDP could reflect a province’s economic prosperity, which may have implications for health infrastructure. A province’s financial capacity could impact resource allocation towards health infrastructure.

### 3.3. Model Setting

A combination of single equation models and simultaneous equation models (SEMs) is utilized in this study to explore the relationships among fiscal decentralization, local government debt, and public health. These mathematical models serve distinct analytical roles, with a more comprehensive and accurate examination of these relationships being offered by SEMs. This enhanced scrutiny arises from two main factors. Firstly, the impact of correlations among individual equations on regression outcomes is not considered in single equation models. For example, an influence on local government debt is exerted by fiscal decentralization while, reciprocally, the shape of fiscal decentralization can be altered by debt through changes in resource distribution and local government behavior. Secondly, biases in estimation results can emerge due to the interconnectedness of independent variables and error terms within the model, when single equation models are used.

In light of these considerations, the two-stage least squares (2SLS) estimator is employed in the SEM to examine the interrelationships among the equations, enhancing the robustness of the estimates. SEM is selected as the benchmark estimation method, as opposed to ordinary least squares (OLS), because biased estimates can be produced by the OLS estimator in the context of potential endogeneity concerns. Consequently, the following equations have been developed:

(15) $${\mathrm{FDE}}_{i,t}=\alpha_{0}+\alpha_{1}GD_{i,t}+\alpha_{2}{\mathrm{PH}}_{i,\;t}+\alpha_{3}{\mathrm{TR}}_{i,t}+\alpha_{4}t+\varepsilon_{i,t}$$

(16) $${\mathrm{FDI}}_{i,t}=\alpha_{0}+\alpha_{1}GD_{i,t}+\alpha_{2}{\mathrm{PH}}_{i,t}+\alpha_{3}{\mathrm{TR}}_{i,t}+\alpha_{4}t+\varepsilon_{i,t}$$

(17) $${\mathrm{PH}}_{i,t}=\beta_{0}+\beta_{1}GD_{i,t}+\beta_{2}{\mathrm{FDE}}_{i,t}+\beta_{3}{\mathrm{MS}}_{i,t}+\beta_{4}\mathrm{DM}+\beta_{5}t+\mu_{i,t}$$

(18) $${\mathrm{PH}}_{i,t}=\beta_{0}+\beta_{1}GD_{i,t}+\beta_{2}{\mathrm{FDI}}_{i,t}+\beta_{3}{\mathrm{MS}}_{i,t}+\beta_{4}\mathrm{DM}+\beta_{5}t+\mu_{i,t\;}$$

(19) $${\;\mathrm{GD}}_{i,t}=\gamma_{0}+\gamma_{1}{\mathrm{FDE}}_{i,t}+\gamma_{2}{\mathrm{PH}}_{i,t}+\gamma_{3}{\mathrm{GDP}}_{i,t}+\gamma_{4}t+\eta_{i,t}$$

(20) $${\;\mathrm{GD}}_{i,t}=\gamma_{0}+\gamma_{1}{\mathrm{FDI}}_{i,t}+\gamma_{2}{\mathrm{PH}}_{i,t}+\gamma_{3}{\mathrm{GDP}}_{i,t}+\gamma_{4}t+\eta_{i,t}$$

The explanation of variables can be found in Table 1, where the designations *i* and *t* refer to the province and the year, respectively. The error terms in three distinct equations are represented by *ε*, *μ*, and η. These equations encompass a range of independent variables, such as fiscal transparency, density of medical institutions, medical staff density, GDP, and population, all of which influence the primary variables. The objective of the models is to quantify the degree to which these factors impact fiscal decentralization, local government debt, and public health, while concurrently addressing potential endogeneity and unobserved factors using methods like the two-stage least squares (2SLS) approach.

## 4. Results and Discussion

### 4.1. Descriptive statistics

Table 2 presents the statistics for fiscal decentralization (FD), local government debt (GD), and public health (PH). The average fiscal decentralization expenditure is 7.386, indicating higher provincial expenditure compared to central expenditure. Public health (PH), gauged by perinatal mortality, averages at 5.094, with an extensive range from 1.44 to 16.9, highlighting pronounced disparities in public health outcomes among provinces. All variables display a coefficient of variation (CV) exceeding 1, implying that the volatility of the data is comfortably within acceptable boundaries. Such an observation underscores that the indicators mentioned earlier do not exhibit an intense polarization in the Chinese context. The relatively low standard deviation values further suggest that significant heteroscedasticity will not be a substantial concern in the ensuing regression analysis. Given that the absolute values for skewness fall below 3 and kurtosis values are under 10, it can be posited that the data are essentially normally distributed. This fulfills the prerequisite of unbiasedness intrinsic to multiple regression analysis.

#### 4.1.1. Correlation Analysis

As shown in Table 3, the analysis of the results demonstrates that the majority of the associations among FDE, FDI, GD, and PH display a strong correlation. In addition, the control variables selected exhibit a significant correlation with the four aforementioned variables, thereby validating the effectiveness of the variable selection process and confirming the foundational presence of the correlation relationships.

#### 4.1.2. Variance Inflation Factor Test

As shown in Table 4, the variance inflation factor (VIF) values, in conjunction with the mean VIF values, all exceed 2, signaling that multicollinearity does not present a severe issue among the variables under consideration.

#### 4.1.3. Smoothing Test

To mitigate the risk of spurious regression in model estimation and to ensure that significant bias is not inherent in the experimental results, a combination of two distinctive tests is employed, Levin, Lin, and Chu test (LLC) and Augmented Dicky–Fuller test (ADF). The outcomes of these tests are presented in Table 5. All variables pass both tests. Consequently, it is appropriate to proceed with the establishment of a PVAR model.

### 4.2. OLS Regression

Table 6 presents the results of the OLS regression analysis. The variable FDE is negatively affected by GD, indicating that, for each unit increase in local government debt, fiscal decentralization expenditure decreases by 0.676 units. Conversely, FDE is positively influenced by public health, suggesting that for each unit increase in public health, fiscal decentralization expenditure increases by 1.002 units. Similarly, FDI is positively affected by TR.

### 4.3. 2SLS Regression

The first model in both tables conducts regressions for FDE, while the second model uses FDI as the dependent and independent variables. Table 7 illustrate various relationships between fiscal decentralization, local government debt, and public health, utilizing FDE and FDI coefficients. An increase in debt corresponds to a decrease in fiscal decentralization, whether measured through expenditure or income. While public health positively correlates with fiscal expenditure decentralization, it shows no significant association with fiscal decentralized expenditure. The public health equation indicates worsening health outcomes with increased debt. Lastly, Table 7 reveals a significant negative impact of fiscal expenditure decentralization on government debt, with a unit increase in FDE leading to a 0.088 unit decrease in debt.

### 4.4. The Bidirectional Relationship between Fiscal Decentralization and Local Government Debt

Implications of Local Government Debt on Fiscal Decentralization: Elevated local government debt necessitates a strategic reallocation of resources, with priority given to debt service. This situation limits the financial autonomy of local governments and impacts fiscal decentralization. Empirical evidence substantiates this proposition, demonstrating a reduction in fiscal decentralization associated with an increase in local government debt, irrespective of whether measured by expenditure or income.

Effect of Fiscal Decentralization on Local Government Debt: Fiscal decentralization, involving the transfer of fiscal responsibilities from central to local governments, incorporates the ability to generate revenue as well as the obligation to allocate funds. In situations where fiscal responsibilities surpass revenue-raising capacities, local governments accumulate debt to fulfill their obligations. This study affirms this, illustrating a significant impact of FDE and FDI on local government debt. Hypothesis 1 is thus confirmed based on empirical results.

### 4.5. The Bidirectional Relationship between Public Health and Fiscal Decentralization

The SEM regression analysis from Table 7 shows a negative association between improvements in public health and fiscal decentralization income. This negative relationship may indicate a reduced reliance on fiscal decentralization for income generation as health outcomes improve, emphasizing the potential of public health as a priority over revenue generation.

The relationship between fiscal decentralization and public health suggests a cyclical pattern. Improvements in public health could lead to a decrease in FDI, potentially demonstrating a reallocation of resources towards enhancing health infrastructure and services. Conversely, increased fiscal decentralization, providing local governments greater control over resources, contributes to public health improvements through the implementation of tailored, responsive policies and interventions. Therefore, the available evidence supports Hypothesis 2.

### 4.6. The Bidirectional Relationship between Government Debt and Public Health

Effect of Government Debt on Public Health: The SEM regression results, represented in Table 4 and Table 5, unveil a significant negative correlation between GD and PH when fiscal decentralization is measured by both FDE and FDI. This indicates that a rise in government debt leads to reduced investment in public health infrastructure and services, potentially worsening public health conditions.

Impact of Public Health on Government Debt: Despite the SEM regression outcomes, depicted in Table 4 and Table 5, suggesting a marginal positive relationship between public health and government debt, FGLS regressions disclose a significant positive correlation. This confirms Hypothesis 3. As public health improves, government debt tends to escalate, presumably due to significant investments in public health infrastructure, services, preventive measures, and treatment programs. Therefore, Hypothesis 3 is confirmed based on empirical results.

## 5. Robustness Test

### 5.1. PVAR Model

The panel vector autoregression (PVAR) model, an advantageous fusion of panel data and vector autoregressive models, is capable of addressing the endogeneity issues and lag periods of variables, thereby facilitating the study of variable interactions [53]. To accurately examine these interactions and impacts, this paper employs the PVAR model for a robustness test, analyzing the evolutionary characteristics of fiscal decentralization, local government debt, and public health in China.

#### 5.1.1. Lagging Items Screening

Drawing from the results of the three information criteria—the Bayesian information criterion (BIC), Akaike information criterion (AIC), and Hannan–Quinn information criterion (HQIC)—it can be seen in Table 8 that, across all samples, the lag order that minimizes the values across different criteria is the first order. Consequently, the lag order of the model is established as the second order.

#### 5.1.2. PVAR Evaluation Results

The tabulated results exhibit outcomes derived from a PVAR model. This model explores the temporal relationship between an array of variables, namely, FDE and FDI, GD and PH. “L” and “L2” denote the first and second lags of these respective variables. The corresponding coefficients embody the estimated influence of the lagged independent variables on the dependent variables.

Both FDE and FDI demonstrate significant interrelations in Table 7 and Table 9, although the exact nature of these associations differs due to the employment of distinct models and lagged variables in Table 9. GD, representing local government debt, and PH also underscore a substantial relationship in both tables. In Table 7, GD is negatively correlated with both FDE and FDI, implying a reverse association between government debt and fiscal decentralization. This relationship is sustained in the PVAR model of Table 9, where the influence of lagged GD on public health is discernibly significant.

Regarding PH, both tables exhibit significant correlations with FDE and FDI. In Table 7, PH presents a negative correlation with FDI, suggesting an inverse relationship between public health and fiscal decentralization income. This association is also observed in the PVAR model, where lagged PH markedly impacts both FDE and FDI.

The consistency of these findings across different tables substantiates a sturdy association between fiscal decentralization, local government debt, and public health. These findings retain their validity across various methodological approaches.

#### 5.1.3. Impulse-Response Functions

**Figure 1:**
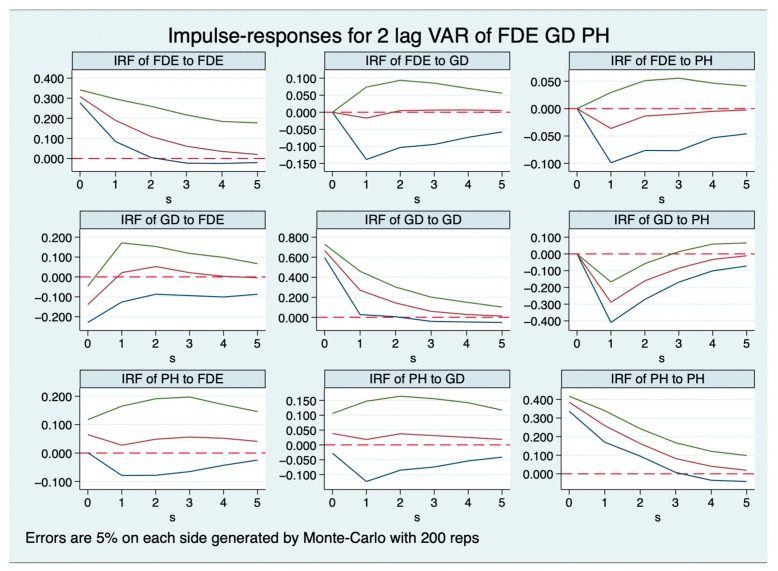
Impulse-response functions with FDE.

**Figure 2:**
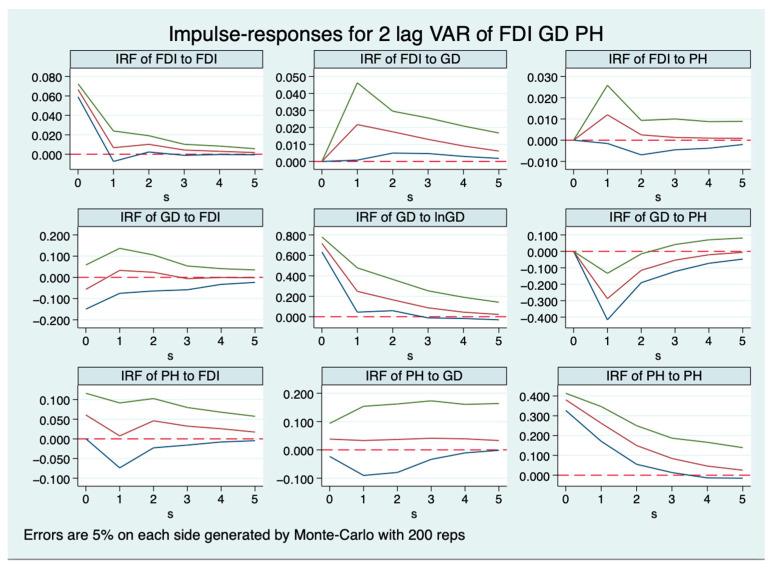
Impulse-response functions with FDI.

Presented in Figure 1 and Figure 2 are the impulse-response functions (IRFs) of FDI, FDE, PH, and GD with a 5% margin of error. The impulse response plots are characterized by three lines: the central line signifies the actual impulse response effect manifested by each endogenous variable in response to a shock, while the upper and lower lines collectively delineate a 95% confidence interval. In Figure 1, the impulse response of FDE to GD is found to be negligible, approaching zero, whereas its response to PH is negative. The impulse response of GD on FDE initially exhibits a negative trajectory, later transitions to positive, and eventually converges to zero. A negative impulse is witnessed from GD to PH, while the impulse response of PH to both FDE and GD is positive.

In Figure 2, a decidedly positive trend is observed in the impulse response of FDI to GD. Moreover, the impulse response of FDI to PH is positive. Analogous to the results in Figure 1, the impulse response of GD to FDI starts off as negative, subsequently turns positive, and finally gravitates towards zero, while a negative impulse is seen from GD to PH. Consistent with the observations in Figure 1, the impulse response of PH to both FDI and GD is positive.

#### 5.1.4. Model Stability Test

As Table 10 indicates, the explanatory capacity of the logarithm of GD and PH for FDE is comparatively weak. Notwithstanding, a steady escalation in the explanatory power of PH over GD is observed, displaying a considerable degree of elucidation. PH is interpreted by both FDE and GD, with a predominant explanatory capacity presented by FDE. Table 11 reveals that FDI is chiefly elucidated by GD, with the interpretive power of GD progressively amplifying and maintaining a certain plateau. Parallel to the insights from Table 10, the explanatory potency of PH on GD persistently intensifies, showcasing a substantial level. PH is elucidated by both FDE and GD, rendering equivalent levels of explanatory potency for FDE and GD.

### 5.2. FGLS Regression

Table 12 presents the results of the feasible generalized least squares (FGLS) regressions. FGLS, a sophisticated variant of OLS, enables efficient estimation in the presence of heteroskedasticity or autocorrelation. A comparison between FGLS and OLS confirms the robustness of the results. For both FDE and FDI models, FGLS indicates a significant negative correlation between local government debt and fiscal decentralization, thereby validating the OLS findings. The models for public health suggest that local government debt has a negative impact on public health, while fiscal decentralization has a positive influence, with the FDE model yielding significant results. The models for government debt indicate a positive correlation between fiscal decentralization and government debt, a finding supported by both FGLS and OLS. The FGLS and OLS results for the GD models reveal a significant and positive relationship between fiscal decentralization and government debt, implying that increased fiscal decentralization leads to an increase in government debt.

## 6. Spatial Heterogeneity Test

As shown in Table 13, given the potential spatial implications of fiscal decentralization, government debt, and public health, this study integrates geographical and economic factors. The methodology involves the adoption of a geoeconomics composite matrix to formulate a spatial panel model. In light of the Moran’s index results, the rejection of the null hypothesis is viable only in the context of FDI and PH. Hence, further analysis is rigorously concentrated on the spatial ramifications associated with FDI and PH.

### Spatial Effect Analysis

Table 14 shows the results of spatial Durbin model, the Lagrange multiplier (LM) test results presented in Table A1 demonstrate partial rejection of the null hypothesis across the three models. This finding signifies the presence of consequential spatial error terms and spatial lag terms within the model, leading to the adoption of the spatial Durbin model (SDM) that encapsulates both these terms for subsequent analysis. The evaluation of results from Table A2, through the lens of the Hausman test, reveals a consistent acceptance of the null hypothesis. This supports the inference that the random-effects model represents the optimal model. A detailed analysis of the SDM model results reveals no significant implications in the spatial effects segment, thereby eliminating the presence of remarkable spatial effects.

The Hausman test results derived from Table A3 demonstrate a rejection of the null hypothesis for the initial model and acceptance for the models thereafter. These findings suggest that the fixed-effects model and the random-effects model represent the optimal models. The SDM model’s results reveal no significant implications within the spatial effects segment, specifically regarding WFDE and WGD, thereby indicating an absence of significant spatial effects.

The Hausman test results from Table A4 show the rejection of the null hypothesis for the first two models but acceptance for the subsequent models, implying that the optimal models are the fixed-effects model and the random-effects model. The SDM model analysis further reveals the spatial effects section’s insignificance, specifically concerning WFDI and WGD, which suggests the absence of substantial spatial effects.

Consequently, this study conclusively negates the presence of spatial effects.

## 7. Conclusions and Discussion

The empirical findings from this study corroborate burgeoning literature emphasizing the intricate relationship between fiscal decentralization and local government debt. An elevated debt burden of local governments undeniably curtails their financial autonomy, leading to a measurable decrease in fiscal decentralization. This resonates with prior studies [54] that illustrate a reduction in fiscal decentralization resulting from escalated local government debt, irrespective of the measurement parameters employed, be it expenditure or income. Conversely, fiscal decentralization, epitomizing the transference of fiscal responsibilities from central to local governments, does precipitate local government debt. This transpires when the magnitude of fiscal responsibilities outstrips the revenue generation capacity, compelling local governments towards borrowing. The empirical results confirm this phenomenon, wherein an amplification in fiscal decentralization provokes local government debt, thereby endorsing Hypothesis 1, aligning with existing studies [16]. This study sheds light on the complex relationship between public health and fiscal decentralization. In opposition to the conventional belief [28] that fiscal decentralization augments public health outcomes, the empirical evidence from the SEM regression analysis presented in Table 7 uncovers a negative association between enhancements in public health and fiscal decentralization income. This underscores a palpable shift away from dependency on fiscal decentralization for revenue generation towards improving public health outcomes. Additionally, the cyclical pattern observed between fiscal decentralization and public health corroborates Hypothesis 2 and aligns with the extant body of research [41]. Enhanced public health conditions lead to a decrease in FDI, indicative of a reallocation of resources towards health infrastructure and service improvement. Inversely, augmented fiscal decentralization, which empowers local governments with greater control over resources, does contribute to public health improvements through the implementation of targeted, responsive policies. Local government debt negatively impacts public health investment, leading to a deterioration in public health conditions.

This research has shed light on the intricate dynamics that connect fiscal decentralization, local government debt, and public health by employing both single equation models and simultaneous equation models. The findings suggest that fiscal decentralization contributes to a decrease in local government debt. However, when it comes to public health outcomes, an increase in local government debt is primarily associated with a decline in health outcomes, indicating that debt is affecting the availability of resources for health services.

Interestingly, this study reveals that increases in medical resources, such as the density of medical institutions and medical staff, do not necessarily lead to improved public health outcomes. This finding challenges conventional wisdom and highlights the need for further research on the efficient allocation and utilization of medical resources. The study emphasizes the importance of prudent fiscal management at the local level, particularly in the context of fiscal decentralization. Furthermore, it underscores the need for targeted and effective health policies that take into account the fiscal context and realities of local governments.

## Figures and Tables

**Table 1:** Variable definition.

Variable	Variable Definition	Description	Data Source
FDE	Per capita fiscal decentralized expenditure	Province fiscal expenditure per capita/central fiscal expenditure per capita	Csmar database
FDI	Per capita fiscal decentralized income	Province fiscal income per capita/central fiscal income per capita	Csmar database
GD	Local government debt	New add special bonds plus one takes logarithm	China electronics local government bonds market access
PH	Public health	Perinatal mortality	Yearbook of health in China
TR	Fiscal transparency	Fiscal transparency index (%)	Research report on fiscal transparency of municipal governments in China
t	Year dummy	Year	
MS	Density of medical institutions	Number of medical beds per 1000 people	Yearbook of health in China
DM	Medical staff density	Number of medical staff per 1000 people	Yearbook of health in China
GDP	Gross domestic product at province level	Log GDP per province	National bureau of statistics of China

**Table 2:** Descriptive statistics.

Variable	N	Mean	SD	Min	Max	CV	Skewness	Kurtosis
FDE	217	7.386	3.962	3.796	24.335	0.536	2.475	9.609
FDI	217	1.287	0.971	0.535	4.996	0.755	2.527	8.65
GD	217	5.241	1.807	0	8.068	0.345	−0.711	2.876
PH	217	5.094	2.588	1.44	16.9	0.508	2.383	9.805
TR	217	0.513	0.158	0.035	0.894	0.309	0.328	2.933
MS	217	5.942	0.952	4.02	8.34	0.16	0.094	2.277
DM	217	6.998	1.38	4.4	13.2	0.197	1.389	6.959
GDP	217	9.556	1.131	6.489	12.533	0.118	−0.397	3.707

**Table 3:** Results of correlation analysis.

	FDE	FDI	GD	PH	TR	MS	DM	GDP
FDE	1							
FDI	0.458 ***	1						
GD	−0.308 ***	0.095	1					
PH	0.549 ***	−0.262 ***	−0.498 ***	1				
TR	0.057	0.655 ***	0.425 ***	−0.471 ***	1			
MS	−0.206 ***	−0.243 ***	0.361 ***	−0.129 *	−0.007	1		
DM	0.190 ***	0.529 ***	0.397 ***	−0.310 ***	0.527 ***	0.398 ***	1	
GDP	−0.423 ***	0.361 ***	0.517 ***	−0.635 ***	0.480 ***	0.092	0.367 ***	1

Note: *** indicate significance at the 1% levels. * indicate significance at the 10% levels.

**Table 4:** Results of variance inflation factor test.

Variable	VIF	Variable	VIF	Variable	VIF	Variable	VIF	Variable	VIF
PH	1.49	DM	1.57	PH	1.99	DM	2.47	GDP	1.8
GD	1.42	GD	1.47	GDP	1.7	FDI	2.1	PH	1.68
TR	1.37	FDE	1.34	FDE	1.45	MS	1.87	FDI	1.15
		MS	1.34			GD	1.26		
Mean	1.43	Mean	1.43	Mean	1.71	Mean	1.93	Mean	1.54

**Table 5:** Results for smoothing test.

Variable	Level	LLC	ADF
FDE	lag(0)	−34.986 ***	5.623 ***
FDI	lag(0)	−23.247 ***	7.931 ***
GD	lag(0)	−22.061 ***	12.131 ***
PH	lag(0)	−17.114 ***	8.955 ***
TR	lag(0)	−32.837 ***	13.677 ***
MS	lag(0)	−32.549 ***	3.852 ***
DM	lag(0)	−39.526 ***	4.654 ***
GDP	lag(0)	−1.856 **	36.223 ***

Note: ***, and ** indicate significance at the 1%, and 5% levels, respectively.

**Table 6:** OLS regression results.

Variables	Model 1 FDE		Model 2 FDI	
	Estimation	SE	Estimation	SE
FDE			FDI	
GD	−0.676 ***	0.220	−0.046	0.037
PH	1.002 ***	0.163	0.016	0.019
TR	11.490 ***	(1.882)	5.037 ***	0.502
_cons	−0.071	338.900	−1.134 ***	0.311
Year	Yes		Yes	
R^2^	0.478		0.525	
F	27.193 ***		34.234 ***	
PH			PH	
GD	−0.446 ***	0.116	−0.931 ***	0.204
FDE	0.380 ***	0.049		
FDI			0.365 **	0.140
MS	0.555 ***	0.182	−0.179	0.228
DM	−0.861 ***	0.090	−0.265	0.161
Year	Yes		Yes	
_cons	7.347 ***	1.626	13.362 ***	2.585
R^2^	0.565		0.345	
F	61.530 ***		18.100 ***	
GD			GD	
FDE	−0.049 **	0.020		
FDI			−0.125 *	0.071
PH	−0.075 *	0.039	−0.115 ***	0.038
GDP	0.519 ***	0.107	0.575 ***	0.108
Year	Yes		Yes	
_cons	1.019	1.204	0.491	1.195
R^2^	0.687		0.683	
F	63.952 ***		52.638 ***	
N	217		217	

Robust standard errors in parentheses. * *p* < 0.1, ** *p* < 0.05, *** *p* < 0.01.

**Table 7:** 2SLS regression with FDE.

Variables	Model 1 FDE		Model 2 FDI	
	Estimation	SE	Estimation	SE
FDE			FDI	
GD	−3.160 **	1.425	−0.995 *	0.511
PH	0.098	0.858	−0.538 *	0.308
TR	11.151 ***	0.024	3.259 **	1.344
Year	Yes		Yes	
_cons	21.913	14.507	8.565	5.206
R^2^	−0.047		−1.243	
PH			PH	
GD	−3.118 **	1.281	−1.661 ***	0.009
FDE	−0.486	0.476		
FDI			−0.894 *	0.537
MS	−0.842	0.827	−0.473	0.405
DM	0.122	0.575	0.094	0.392
Year	Yes		Yes	
_cons	33.049 **	12.845	18.883 ***	546.800
R^2^	−1.194		0.178	
GD			GD	
FDE	−0.088 **	0.039		
			−0.285**	0.135
PH	−0.269 *	0.163	−0.416**	0.200
GDP	0.185	0.259	0.200	0.273
Year	Yes		Yes	
_cons	6.576 *	3.349	6.810 *	3.611
R^2^	0.623		0.573	
*N*	217		217	

Robust standard errors in parentheses. * *p* < 0.1, ** *p* < 0.05, *** *p* < 0.01.

**Table 8:** Lagging items screening.

	FDE			FDI		
Lag	AIC	BIC	HQIC	AIC	BIC	HQIC
1	5.934	7.936	6.746	3.207	5.210	4.020
2	5.235 *	7.760 *	6.261 *	2.287 *	4.811 *	3.312 *
3	5.574	8.842	6.893	5.319	8.587	6.638
4	7.308	11.733	9.045	5.460	9.886	7.197
5	32.245	38.629	34.326	23.119	29.502	25.200

* *p* < 0.1.

**Table 9:** PVAR evaluation results.

	FDE			FDI		
	FDE	GD	PH	FDE	GD	PH
L.FDE	0.626 ***	0.429	−0.057			
	(0.221)	(0.286)	(0.241)			
L.FDI				0.096	1.514 *	−0.508
				(0.149)	(0.890)	(0.744)
L.GD	−0.020	0.447 **	−0.011	0.029	0.388 **	0.009
	(0.100)	(0.181)	(0.122)	(0.021)	(0.161)	(0.103)
L.PH	−0.094	−0.749 ***	0.673 ***	0.031	−0.755 ***	0.689 ***
	(0.107)	(0.194)	(0.113)	(0.022)	(0.228)	(0.135)
L2.FDE	−0.022	−0.138	0.162			
	(0.139)	(0.174)	(0.119)			
L2.FDI				0.131 *	−0.269	0.742 ***
				(0.068)	(0.348)	(0.238)
L2.GD	0.030	0.039	0.043	0.010 **	0.061	0.035
	(0.022)	(0.065)	(0.027)	(0.005)	(0.060)	(0.029)
L2.PH	0.072	0.463 ***	−0.045	0.003	0.463 ***	−0.058
	(0.071)	(0.165)	(0.130)	(0.014)	(0.164)	(0.125)

Note: ***, **, and * indicate significance at the 1%, 5%, and 10 % levels, respectively.

**Table 10:** Model stability test (FDE).

s	FDE			GD			PH		
	FDE	GD	PH	FDE	GD	PH	FDE	GD	PH
1	1	0	0	0.042	0.958	0	0.027	0.01	0.963
2	0.988	0.002	0.01	0.032	0.834	0.134	0.022	0.008	0.969
3	0.988	0.002	0.01	0.034	0.804	0.162	0.029	0.013	0.958
4	0.987	0.002	0.011	0.034	0.796	0.17	0.04	0.016	0.944
5	0.987	0.003	0.011	0.034	0.795	0.172	0.049	0.018	0.933
6	0.986	0.003	0.011	0.034	0.795	0.172	0.055	0.019	0.926
7	0.986	0.003	0.011	0.034	0.795	0.172	0.058	0.02	0.922
8	0.986	0.003	0.011	0.034	0.795	0.172	0.06	0.02	0.92
9	0.986	0.003	0.011	0.034	0.795	0.172	0.06	0.02	0.919
10	0.986	0.003	0.011	0.034	0.795	0.172	0.061	0.02	0.919

**Table 11:** Model stability test (FDI).

	FDI			GD			PH		
s	FDI	GD	PH	FDI	GD	PH	FDI	GD	PH
1	1	0	0	0.006	0.994	0	0.025	0.01	0.966
2	0.879	0.093	0.028	0.006	0.869	0.125	0.017	0.012	0.971
3	0.832	0.141	0.027	0.007	0.857	0.137	0.024	0.016	0.96
4	0.808	0.166	0.027	0.007	0.855	0.139	0.027	0.022	0.951
5	0.796	0.178	0.026	0.007	0.854	0.139	0.029	0.027	0.943
6	0.791	0.183	0.026	0.007	0.855	0.139	0.03	0.031	0.938
7	0.789	0.185	0.026	0.007	0.855	0.139	0.031	0.034	0.936
8	0.788	0.186	0.026	0.007	0.855	0.139	0.031	0.035	0.934
9	0.787	0.186	0.026	0.007	0.855	0.139	0.031	0.036	0.933
10	0.787	0.186	0.026	0.007	0.855	0.139	0.031	0.036	0.933

**Table 12:** FGLS regression results.

Variables	Model 1		Model 2	
	Estimation	SE	Estimation	SE
FDE			FDI	
GD	−0.676 ***	0.216	−0.046	0.036
PH	1.002 ***	0.160	0.016	0.018
TR	11.490 ***	1.843	5.037 ***	0.492
Year	Yes		Yes	
_cons	1.484	1.995	−1.003 ***	0.345
PH			PH	
GD	0.446 ***	0.113	−0.931 ***	0.199
FDE	0.380 ***	0.048		
			−0.365 ***	0.136
MS	0.555 ***	0.178	−0.179	0.223
DM	−0.861 ***	0.087	−0.265 *	0.157
Year	Yes		Yes	
_cons	7.923 ***	1.891	14.409 ***	3.032
GD			GD	
FDE	−0.049 **	0.020		
			2.215 *	1.214
PH	−0.075 *	1.193	−0.115 ***	0.037
GDP	0.519 ***	0.105	0.575 ***	0.106
Year	Yes		Yes	
_cons	2.153 *	1.287	14.409 ***	3.032
N	217		217	

Robust standard errors in parentheses. * *p* < 0.1, ** *p* < 0.05, *** *p* < 0.01.

**Table 13:** Moran’s index results.

Year	FDI		FDE		GD		PH	
	Z	*p*	Z	*p*	Z	*p*	Z	*p*
2015	5.7653	0.000 ***	0.7189	0.4722	−0.9542	0.34	2.2468	0.0247 **
2016	5.8157	0.000 ***	0.8829	0.3773	1.6919	0.0907 *	1.7134	0.0866 *
2017	5.8453	0.000 ***	0.845	0.3981	1.8125	0.0699 *	2.0388	0.0415 **
2018	5.7772	0.000 ***	0.7964	0.4258	1.3981	0.1621	2.1679	0.0302 **
2019	5.889	0.000 ***	0.6685	0.5038	1.3283	0.1841	2.4321	0.015 **
2020	5.775	0.000 ***	0.432	0.6658	0.7632	0.4454	2.3081	0.021 **
2021	5.7871	0.000 ***	0.582	0.5606	0.2226	0.8239	2.2898	0.022 **

* *p* < 0.1, ** *p* < 0.05, *** *p* < 0.01.

**Table 14:** Results of spatial Durbin model.

SDM(RE)		SDM(FE)		SDM(FE)	
FDI		PH		PH	
WlnGD	−0.0109	WFDE	−0.4318	WFDI	−1.2980
	(0.0187)		(0.4078)		(1.2656)
WPH	−0.0454	WlnGD	0.1134	WlnGD	0.1196
	(0.0353)		(0.1265)		(0.1271)
WTR	−0.1883	WMS	−2.6300 ***	WMS	−2.8375 ***
	(0.2502)		(0.7674)		(0.8025)
		WDM	0.9276 *	WDM	1.0329 **
			(0.4765)		(0.5206)

Robust standard errors in parentheses. * *p* < 0.1, ** *p* < 0.05, *** *p* < 0.01.

## Data Availability

The data presented in this study are available on request from the corresponding author.

## Appendix A

**Table A1:** Lagrange multiplier (LM) test results.

Model	FDI = GD + PH + TR		PH = FDE + GD + MS + DM		PH = FDI + GD + MS + DM	
	Statistic	*p*	Statistic	*p*	Statistic	*p*
Spatial error: Lagrange multiplier	35.004	0.000 ***	0.858	0.354	1.477	0.224
Spatial error: robust Lagrange multiplier	1.485	0.223	2.27	0.132	5.247	0.022 **
Spatial lag: Lagrange multiplier	40.422	0.000 ***	5.248	0.022 **	0.006	0.938
Spatial lag: robust Lagrange multiplier	6.903	0.009 ***	6.66	0.01 **	3.776	0.052 *

Robust standard errors in parentheses. * *p* < 0.1, ** *p* < 0.05, *** *p* < 0.01.

**Table A2:** The spatial Durbin model (SDM) revaluation result.

	SDM(RE)	SAR(RE)	SEM(RE)
	FDI	FDI	FDI
GD	−0.0103	−0.0098	−0.0126
	(0.0104)	(0.0072)	(0.0085)
PH	0.0246 *	0.0200 *	0.0256 **
	(0.0130)	(0.0117)	(0.0123)
TR	0.3262 **	0.2547 **	0.2866 **
	(0.1399)	(0.1126)	(0.1289)
_cons	0.7276 **	0.4302 *	1.0758 ***
	(0.3126)	(0.2363)	(0.2032)
WGD	−0.0109		
	(0.0187)		
WPH	−0.0454		
	(0.0353)		
WTR	−0.1883		
	(0.2502)		
rho	0.5721 ***	0.5539 ***	
	(0.1220)	(0.1231)	
lambda			0.4738 ***
			(0.1401)
lgt_theta	−2.8420 ***	−2.8497 ***	
	(0.1497)	(0.1492)	
sigma2_e	0.0145 ***	0.0147 ***	0.0147 ***
	(0.0015)	(0.0015)	(0.0015)
ln_phi			4.1249 ***
			(0.2779)
*N*	217	217	217
ll	58.3191	57.1873	54.2990
hau_chi2	4.6747	3.0603	6.4335
hau_chi2_p	0.6996	0.5478	0.1690
r2_w	0.0482	0.0446	0.0585

Robust standard errors in parentheses. * *p* < 0.1, ** *p* < 0.05, *** *p* < 0.01.

**Table A3:** The Hausman test results (FDE).

	SDM(FE)	SAR(RE)	SEM(RE)
	PH	PH	PH
FDE	0.2251 **	0.3323 ***	0.3412 ***
	(0.1104)	(0.0650)	(0.0672)
GD	−0.0069	−0.0650	−0.0683
	(0.0530)	(0.0521)	(0.0692)
MS	0.7381 ***	0.4079 **	0.5798 **
	(0.2297)	(0.1849)	(0.2332)
DM	−0.1344	−0.4671 ***	−0.5339 **
	(0.1976)	(0.1346)	(0.2073)
_cons		1.1357	3.2293
		(1.2674)	(2.2199)
WFDE	−0.4318		
	(0.4078)		
WGD	0.1134		
	(0.1265)		
WMS	−2.6300 ***		
	(0.7674)		
WDM	0.9276 *		
	(0.4765)		
rho	0.3981 ***	0.5468 ***	
	(0.1415)	(0.1165)	
lambda			0.7039 ***
			(0.1889)
sigma2_e	0.3440 ***	0.4541 ***	0.4565 ***
	(0.0332)	(0.0480)	(0.0535)
lgt_theta		−1.7092 ***	
		(0.1693)	
ln_phi			1.8561 ***
			(0.3220)
*N*	217	217	217
ll	−193.5385	−283.3363	−287.0429
hau_chi2	18.7305	4.0618	2.8300
hau_chi2_p	0.0276	0.5406	0.7262
r2_w	0.4771	0.3896	0.2480

Robust standard errors in parentheses. * *p* < 0.1, ** *p* < 0.05, *** *p* < 0.01.

**Table A4:** The Hausman test results (FDI).

	SDM(FE)	SAR(FE)	SEM(RE)
	PH	PH	PH
FDI	0.7751 **	0.5752	0.2537
	(0.3636)	(0.3642)	(0.3048)
GD	−0.0093	−0.0613	−0.0692
	(0.0531)	(0.0492)	(0.0673)
MS	0.8272 ***	0.4437 **	0.7115 ***
	(0.2177)	(0.2074)	(0.2371)
DM	−0.1678	−0.4630 ***	−0.4945 **
	(0.1932)	(0.1490)	(0.2202)
_cons			4.3717 **
			(2.1928)
WFDI	−1.2980		
	(1.2656)		
WGD	0.1196		
	(0.1271)		
WMS	−2.8375 ***		
	(0.8025)		
WDM	1.0329 **		
	(0.5206)		
rho	0.3899 ***	0.5802 ***	
	(0.1417)	(0.1110)	
lambda			0.7603 ***
			(0.1354)
sigma2_e	0.3436 ***	0.3834 ***	0.4462 ***
	(0.0332)	(0.0372)	(0.0507)
ln_phi			2.5572 ***
			(0.3022)
N	217	217	217
ll	−193.3614	−207.3026	−296.4771
hau_chi2	16.4615	10.3903	4.0336
hau_chi2_p	0.0578	0.0649	0.5446
r2_w	0.4783	0.3992	0.1532

Robust standard errors in parentheses. ** *p* < 0.05, *** *p* < 0.01.
